# Barriers and facilitators with medication use during the transition from hospital to home: a qualitative study among patients

**DOI:** 10.1186/s12913-019-4028-y

**Published:** 2019-03-29

**Authors:** Sara Daliri, Charlotte L. Bekker, Bianca M. Buurman, Wilma J. M. Scholte op Reimer, Bart J. F. van den Bemt, Fatma Karapinar – Çarkit

**Affiliations:** 1 Faculty of Health, ACHIEVE Center of Expertise, Amsterdam University of Applied Sciences, Amsterdam, 1105 BD the Netherlands; 2 Department of Clinical Pharmacy, OLVG hospital, Amsterdam, 1061AE The Netherlands; 3 Department of Pharmacy, Sint Maartenskliniek, Nijmegen, 6574 NA The Netherlands; 4 Department of Clinical Pharmacy, Division of Laboratory and Pharmacy, University Medical Centre Utrecht, Utrecht, 3584 CX The Netherlands; 5 Department of Internal Medicine, section of Geriatric Medicine, Academic Medical Center, Amsterdam, 1105 AZ the Netherlands; 6 Department of Cardiology, Academic Medical Center, Amsterdam, 1105 AZ The Netherlands; 7 Department of Pharmacy, Radboud University Medical Centre, Nijmegen, 6525 GA The Netherlands; 8 Department of Pharmacy, University Medical Centre Maastricht, Maastricht, 6229 HX The Netherlands

**Keywords:** Continuity of patient care, Medication errors, Focus groups, Qualitative research

## Abstract

**Background:**

During transitions from hospital to home, up to half of all patients experience medication-related problems, such as adverse drug events. To reduce these problems, knowledge of patient experiences with medication use during this transition is needed. This study aims to identify the perspectives of patients on barriers and facilitators with medication use, during the transition from hospital to home.

**Methods:**

A qualitative study was conducted in 2017 among patients discharged from two hospitals using a semi-structured interview guide. Patients were asked to identify all barriers they experienced with medication use during transitions from hospital to home, and facilitators needed to overcome those barriers. Data were analyzed following thematic content analysis and visualized using an “Ishikawa” diagram.

**Results:**

In total, three focus groups were conducted with 19 patients (mean age: 70.8 (SD 9.3) years, 63% female). Three barriers were identified; lack of personalized care in the care continuum, insufficient information transfer (e.g. regarding changes in pharmacotherapy), and problems in care organization (e.g. medication substitution). Facilitators to overcome these barriers included a personal medication-counselor in the care continuum to guide patients with medication use and overcome communication barriers, and post-discharge follow-up care (e.g. home visits from healthcare providers).

**Conclusions:**

During transitions from hospital to home patients experience individual-, healthcare provider- and organization level barriers. Future research should focus on personal-medication counselors in the care continuum and post-discharge follow-up care as it may overcome communication, emotional, information and organization barriers with medication use.

**Electronic supplementary material:**

The online version of this article (10.1186/s12913-019-4028-y) contains supplementary material, which is available to authorized users.

## Background

Research shows that the transition from hospital to home presents a critical period for patient safety as it can result in medication-related problems (MRPs) post-discharge [1–3]. MRPs are defined as events or circumstances related to a patient’s medication therapy, which can actually or potentially interfere with desired health outcomes [4–6]. The prevalence of MRPs post-discharge varies from 14 to 49% [7]. A recent study showed that a median of 20% of hospital readmissions are due to MRPs, of which 69% were regarded as preventable [8].

There are 4 key problems causing these MRPs in the transition from hospital to home. First, during hospitalization, the hospital staff are responsible for daily management and the intake of patient medication, while patients are expected to self-manage their medication regimen after discharge, often with limited guidance [9]. Second, if instructions on medication changes are provided, this usually takes place at the time of discharge. However, at discharge, patients are often distracted, overloaded with information and eager to leave for home, therefore paying less attention to medication instructions [9]. Third, oral and written instructions are not always adjusted to patients’ literacy levels or informational needs [10, 11]. Consequently, most patients have difficulties implementing the changed regimen in their daily lives [12–14]. Lastly, patients’ healthcare providers in primary care are insufficiently informed regarding the reasons for medication changes and therefore have difficulties monitoring patients’ entire medication regimens [14–16].

A powerful strategy for improving healthcare is the introduction of patient-centered care, because it addresses barriers by responding to patient-specific needs, preferences and values [17–19]. To design effective interventions to reduce MRPs in the transition from hospital to home, healthcare providers and organizations need to understand patients’ perspectives and respond more fully to their needs. This requires an understanding of barriers that patients experience that could result in MRPs but also of facilitators that could reduce MRPs. Until now, several studies have identified patient barriers and facilitators at transitions of care but focused on the hospital discharge process in general rather than the transition from hospital to home [20, 21] or observed post-discharge problems throughout observations from healthcare providers [22]. Few studies have explored patient perspectives, specifically focusing on medication. However, studies that did focus on medication were limited as they studied only a specific topic regarding barriers to medication use (e.g. communication failures) [23], focused primarily on barriers [24], asked patients to reflect upon pre-defined barriers [25] or studied the discharge moment itself rather than the transition from hospital to home [26]. Two studies identified patients’ perceived barriers and facilitators with medication use by using a survey, so were therefore lacking a deeper exploration of the issues [27, 28]. There is still need for an exploration of those factors which patients perceive as barriers and facilitators for the continuity of medication use following hospital discharge. The aim of this study therefore is to identify patients’ perspectives on barriers and facilitators with medication use during the transition from hospital to home.

## Methods

This qualitative study is reported following the Consolidated criteria for reporting qualitative research (COREQ) [29].

### Study design and setting

A qualitative study was conducted in March 2017 with patients from two Dutch hospitals (OLVG and BovenIJ) using focus groups. The study was approved by the local ethics committee “Adviescommissie Wetenschappelijk Onderzoek-Medisch-Ethische Commissie” (ACWO-MEC) OLVG hospital (ID WO: 15.067) and the Board of Directors BovenIJ hospital (ID WO: 5EMeh545).

Focus groups were chosen because this enables an in-depth discussion among patients about their perspectives of barriers and facilitators with medication use during the transition from hospital to home. This qualitative study was part of a previously conducted pharmacy-led transitional care multicenter study in the Netherlands, which took place in the OLVG and BovenIJ hospitals and 50 community pharmacies [30]. The intervention of this study consisted of medication reconciliation during hospitalization, including ‘teach-back’ at hospital discharge to check whether medication changes had been clearly explained to patients. Also, primary healthcare providers received an overview listing reasons for in-hospital medication changes and the community pharmacist visited the patient at home within 5 days post-discharge.

### Participants

This study focused on patients discharged from the departments of internal medicine, cardiology, pulmonology or neurology. Patients who received the intervention from the pharmacy-led transitional care multicenter study (*n* = 197, see Table 1 for all inclusion and exclusion criteria) were eligible for participation in the focus groups. Patients included in the first phase of the intervention (*n* = 50) were excluded to limit recall-bias. The participants were all prone to post-discharge MRPs as they had to fulfil inclusion criteria which are associated with the occurrence of MRPs [2, 3]. By selecting this high-risk population, we expect to gain more insight into barriers and facilitators, as patients are more likely to experience challenges during transitions of care and know better what is needed to overcome these problems. Furthermore, the intervention group was specifically chosen because previous research showed that patient have difficulties to address their needs when they do not know how care can be organized [10]. Of the 147 eligible patients, 125 (85.0%) were contacted by phone for participation. The remaining patients were either deceased (*n* = 6) or had withdrawn from the multicenter study (*n* = 16). Contacted patients received oral study information and 37 patients were interested in participating. Of these, 24 patients randomly received a confirmation letter by post. Prior to every focus group, written informed consent was obtained from each participant, ensuring anonymity and confidentiality of the information obtained.

**Table 1:** Patient inclusion and exclusion criteria [30]

Inclusion criteria	Exclusion criteria
˗ Discharge from department: internal medicine, cardiology, pulmonology or neurology ˗ ≥24 h hospital admission ˗ Use of ≥3 chronic prescription medications at discharge ˗ ≥1 change in medication regimen (intended for chronic use) conducted during hospitalization	˗ Discharge to another institution, e.g. rehabilitation center or nursing home ˗ Patients who could not be counselled, as stated by hospital physicians or nurses, due to physical/mental constraints, language restrictions or terminal illness ˗ Patients included in the first phase of the intervention

### Interview guide

A semi-structured interview guide was derived from discussion with a multi-disciplinary panel of clinicians, patient representatives and researchers, and consisted of 2 questions about (1) patients’ experiences on barriers with medication use during transitions from hospital to home and (2) patients’ suggested facilitators on how to overcome those barriers. Patients could stress any barriers they had experienced during any transition from hospital to home or reflect on those barriers that were experienced by others, e.g. relatives or friends. The interview guide was based on previous research of researchers who have over 10 years’ experience with transitional care and medication-related problems due to transitions in care. This included interviews with patients on their informational needs before and after discharge and surveys to assess MRPs [10, 26].

### Data collection

A trained focus group moderator who was not involved in the pharmacy-led transitional care multicenter study moderated the focus groups using the interview guide. A list with examples of barriers and facilitators with medication use during transitions from hospital to home was developed which the moderator could use in case participants needed help (Additional file 1). Also, the first researcher (XX) assisted the moderator if necessary, and observed and made field notes during each focus group. All focus groups were held in a hospital meeting room and lasted from 1.5 to 2 h. After participation, patients were rewarded with a 25 euro gift voucher. Focus groups were audio recorded and transcribed verbatim. To ensure correct interpretation, member checking was done for all the focus groups.

### Data analysis

Transcripts were systematically analyzed using a thematic-content approach in the software program MAXQDA version 12. This comprised the initial generation of codes, which were subsequently compared and grouped into themes, followed by a thorough review of the themes. First, two researchers (XX and YY) independently analyzed, compared and discussed the coding of the first transcript until consensus was achieved. Hereafter, the first researcher (XX) coded the other transcripts, which was completely reviewed by the second researcher (YY). Also, any differences were discussed here. Both researchers placed the codes into categories and subsequently into sub- and main themes. Finally, the themes were thoroughly discussed in research group meetings until a consensus was reached. Once the final themes were identified, “Ishikawa” diagrams (i.e. fishbone diagrams) were created to illustrate the identified barriers and facilitators [31]. This fishbone diagram is a structured tool to understand contributing factors that lead to an effect or problem. Representative quotes were selected for each theme.

## Results

In total, 3 focus groups were conducted with 19 patients (3 cancelled due to health issues and 2 did not show up), which lasted between 92 and 124 min. The demographic characteristics of the participants (Additional file 2) show similarities to the characteristics of the complete intervention group population (Table 2).

**Table 2:** Demographic characteristics of focus groups and total intervention group patients

	Group 1 (*n* = 7)	Group 2 (*n* = 7)	Group 3 (*n* = 5)	Overall (*n* = 19)
Age (years)				
Mean ± SD	69.0 [9.3]	76.3 [5.3]	65.6 [11.3]	70.8 [9.3]
Gender				
Male [n, (%)]	4 (57.1)	5 (71.4)	3 (60.0)	12 (63.2)
Number of medications at discharge				
Mean ± SD	9.9 [3.0]	8.4 [3.7]	10.8 [2.3]	9.6 [3.1]
Number of in-hospital medication changes per patient				
Mean ± SD	2.9 [1.3]	3.6 [2.0]	4.2 [2.6]	3.5 [1.9]
Help with medication use at home (e.g. homecare) [n, (%)]	1 (14.3)	2 (28.6)	0 (0)	3 (15.8)
Living situation [n, (%)]				
Living alone	2 (28.6)	3 (42.9)	1 (20.0)	6 (31.6)
Living together	5 (71.4)	4 (57.1)	4 (80.0)	13 (68.4)
Hospital [n, (%)]				
BovenIJ	6 (85.7)		1 (20.0)	7 (36.8)
OLVG	1 (14.3)	7 (100.0)	4 (80.0)	12 (63.2)
Admission type [n, (%)]				
Unplanned	5 (71.4)	7 (100.0)	5 (100.0)	17 (89.5)
Planned	2 (28.6)			2 (10.5)
Ward type [n, (%)]				
Cardiology	4 (57.1)	4 (57.1)	2 (40.0)	10 (52.6)
Internal Medicine	2 (28.6)	2 (28.6)	2 (40.0)	6 (31.6)
Pulmonology		1 (14.3)	1 (20.0)	2 (10.5)
Neurology	1 (14.3)			1 (5.3)

During the focus groups, the example list of barriers and facilitators (Additional file 1) was not used by the moderator because there was a lot of discussion among participants, as they reacted on each others experiences, especially when they had to think of facilitators. In total, 3 main themes of barriers and 2 main themes of facilitators were identified.

### Barriers with medication use during the transition from hospital to home

**Fig. 1:**
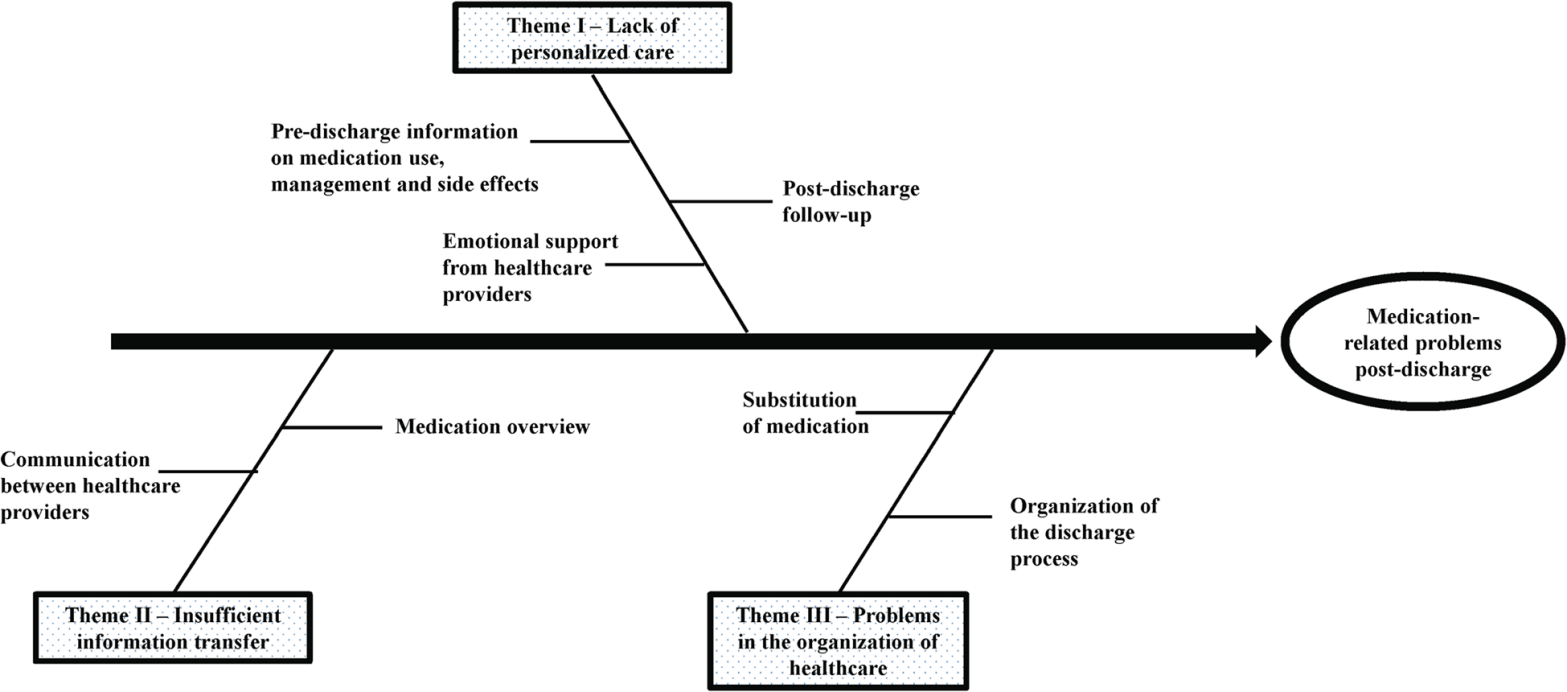
Fishbone diagram of patient-reported barriers to medication use during the transition from hospital to home

The identified themes and their subthemes are presented in Fig. 1. In total, 3 main themes of barriers were identified:

(I) *Lack of personalized care*, categorized into 3 subthemes: (a) pre-discharge information on medication use, management and side effects, (b) emotional support from healthcare providers, and (c) post-discharge follow-up.
(II) *Insufficient information transfer*, categorized into 2 subthemes: (a) communication between healthcare providers and (b) medication overview.
(III) *Problems in the organization of healthcare*, categorized into 2 subthemes: (a) organization of the discharge process and (b) substitution of medication.

### Theme Ι – lack of personalized care

#### Pre-discharge information on medication use, management and side effects

Patients indicate that discharge instructions on how to use and manage medication at home are often not tailored to their personal condition or needs, which can lead to problems in understanding the information given:

*“But I had a brain hemorrhage and I must think thoroughly about something before I can really take it in. I really need that one to one communication and people taking time for me. Even then, it is still doubtful if I really take it in, you know.”(Female, 54 years-old, internal medicine)*

Also, according to several patients, potential treatment-related side effects were rarely discussed with them during hospitalization:

*“Something I think can be improved, and which really did not happen in my case, but may not be the same for everyone, is that they indicate in advance what may be possible side effects of the medication.” (Male, 73 years old, cardiology)*

In contrast, for some patients knowing about all the potential side effects in advance causes harm rather than relief:

*“You may get dizzy if you know, and if you don’t, you won’t.” (Female, 80 years, pulmonology)*

#### Emotional support from healthcare providers

During the focus groups, some patients mentioned that the start of a new medication therapy during their hospital stay can feel overwhelming. They feel that healthcare providers do not always notice the impact of medication changes on a patient’s mental well-being and sometimes miss their emotional support to cope with these changes:

*“In any event, it is quite a shock if you have never used medication and suddenly have to take so many all at once. Then you think, well, this may save me for the time being, but soon I will die from some other cause anyway. And of course they don’t pay attention to that.” (Male, 73 years old, cardiology)*

#### Post-discharge follow-up

Some patients questioned whether healthcare providers assess if patients are capable of using and managing medication independently post-discharge, as patients are expected to self-manage their medication regimen post-discharge. Patients considered especially vulnerable patients, such as the elderly, who are often prescribed many medications, are at a higher risk of improper post-discharge medication use when they do not receive post-discharge guidance.

“*… With regard to the medication, after having been discharged, I also think that they (the healthcare providers) should assess very well whether this lady is able to manage everything on her own. … because … I have also stood up in the morning and thought, alright, that is a bag full of medication. Then I think, okay, well, I will arrange everything neatly, in a relaxed way, but I’m fifty-four.”(Female, 54 years old, cardiology)*

### Theme ΙΙ – insufficient information transfer

#### Communication between healthcare providers

Many patients mentioned that communication between healthcare providers on changes in pharmacotherapy often seems to be lacking. Therefore, patients fear mistakes in their medication; one patient even mentioned that this results in distrust of his healthcare providers:

*“My Metformin was reduced from two to one. Then I visited my GP and had to explain to him that it had been reduced to one. A week later I visited the nurse and I had to recount the whole story all over again. Then, I came to the hospital and the doctor says ‘so you are taking two tablets of Metformin’. ‘No I have been on one tablet for over two months now’. Then I think to myself: Come on guys, if you are making mistakes with that, I wonder what mistakes you are making with my medication. So I don’t have much confidence in this business here.”(Male, 56 years old, cardiology)*

Consequently, some patients feel responsible for an adequate information transfer between healthcare providers and introduce solutions themselves:

*“I did that when I was given a lot of medications, four other medications; I made a complete list for the pharmacist, sending a CC to my GP, because it turns out that my GP does not always, or rather, hardly ever, receives a list from the hospital of the medications that I take.”(Male, 80 years old, cardiology)*

#### Medication overview

At hospital discharge, patients often receive a medication overview, listing their medication. However, according to many patients, this overview is often incomplete when patients do not pay attention:

*“The various medications you get from the hospital are listed, but the rest is what you indicate yourself, so if you forget something, it won’t be on the medication overview either.” (Male, 62 years old, pulmonology)*

### Theme ΙΙΙ – problems in organization of healthcare

#### Organization of the discharge process

Many patients find it frustrating when they have to wait for a long time to get their medication supply, especially when the discharge time is planned beforehand. Some patients mentioned this results in wanting to leave the hospital without getting any medication.

*“At eleven o’clock I could go home and I was so happy, but then I had to wait until four o’clock for my medication. I became cranky about that; I said to my husband, I will go home without my medication, I don’t care what they think.”(Female, 75 years old, internal medicine)*

Furthermore, some patients experience problems in receiving sufficient medication when they are discharged just before the weekend. The supply of medication dispensed at discharge is not always enough to last until refill prescriptions are available from their usual source (e.g. community pharmacy).

#### Substitution of medication

The substitution of medication (e.g. switch from brands to generics), during the transition from hospital to home confuses and sometimes frightens patients. Substituted medication often looks different from the medication they are used to taking and therefore many patients fear using the substitute:

*“I did indeed get medication that looked very different; then, it is really scary to take them. The other (usual) ones you just swallow.” (Female, 75 years old, internal medicine)*

### Facilitators to overcome identified barriers with medication use during the transition from hospital to home

**Fig. 2:**
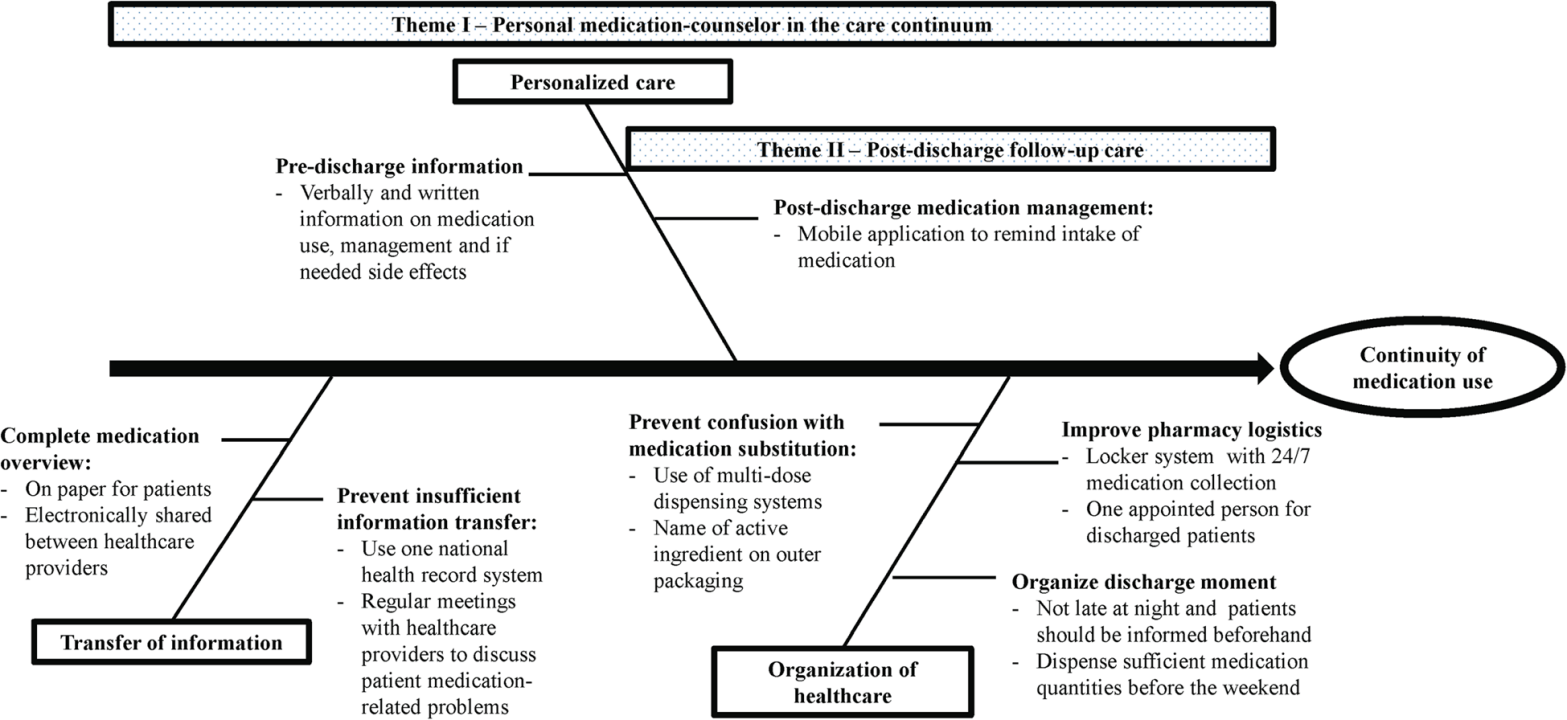
Fishbone diagram of patient-reported facilitators to overcome identified barriers during the transition from hospital to home

Many facilitators were suggested by patients to overcome identified (sub) themes of barriers and are presented in Fig. 2. Some facilitators were mentioned that were not directed at a specific barrier but were generally perceived as helpful, including the improvement of pharmacy logistics. For instance, by introducing a locker service with 24/7 accessibility, which allows patients to collect their medication anytime of the day and skip queues, or to assign one dedicated pharmacy assistant in the pharmacy who is responsible for preparing all the discharge prescriptions.

Overall, patients agreed that facilitators should at least target vulnerable patients and should anticipate their preferences to result in more personalized care.

Also, patients reported that information on medication use, management or side effects, should always be provided both verbally and written, as patients are often distracted during hospitalization and therefore might not comprehend given instructions right away:

*“That is why it is so important to give the oral explanation at the time of discharge, but it also depends on what kind of person it concerns, what his state of mind and condition is. There are people who are actually not well enough to go home, who shouldn’t be discharged, but who are sent home anyway because there is no medical reason to keep them hospitalized. They may have all kinds of worries in their heads. Therefore, it would be good to get a written explanation in addition to the oral one.” (Male, 62 years old, pulmonology)*

Also, according to patients, communication problems among healthcare providers regarding changes in pharmacotherapy could be targeted when healthcare providers from both primary and secondary care organize regular meetings together and discuss patient changes in medication regimens and any encountered MRPs. Patients suggested the use of one national health record system by healthcare providers.

From all of the suggested facilitators, in total, 2 main themes were identified: *personal medication-counsellor in the care continuum* and *post-discharge follow-up care* (Fig. 2) which are further described below. Both types of facilitators could overcome more than one (sub) theme of identified barriers.

### Personal medication-counsellor in the care continuum

Predominantly, all patients were in favor of introducing a personal medication-counsellor for patient-centered medication care in the care continuum. The counsellor could be assigned to every patient who is in need of extra care and could closely monitor patients. Patients agreed that this person could assess a patient’s capability to use and manage medication at home, and discuss potential problems and solutions:

*“If he has a conversation with you within a reasonable period of time, he will see what the weak points are, and then he can discuss with you there and then how to correct these, or say: we will solve it in that way.” (Male, 56 years old, cardiology)*

Patients discussed that this counsellor should focus on their personal issues, preferences and needs and should be able to empathize with the patient:

*“The point is that he is a human being. It is about human beings, after all. It is a human being who is listening to what we have to say, and who actually takes the trouble of finding out hey, this is this gentleman’s problem or that is his or her problem, we’ll try and figure out what is going on here.” (Male, 56 years old, cardiology)*

Overall, patients expressed that the counsellor could act as a point of contact between the patient and the healthcare providers in the care continuum:

*“You are given so much information; you are told this by one and that by another, but part of it just does not fit. There should be one point of contact.” (Male, 78 years old, cardiology)*

Patients agreed that the patient-counsellor should be a professional who is familiar with medication therapies and is able to communicate with patients and other healthcare providers. Some believed that general practitioners, nurses or pharmacists were the best professionals to fulfil this role, because they are more closely related to the patient compared to others. However, patients also acknowledged that this would most likely be an expensive service and therefore probably not feasible.

### Post-discharge follow-up care

Follow-up care after hospital discharge was also seen as an important facilitator to overcome the mentioned barriers during the transition from hospital to home.

For instance, many patients agreed that a simple phone call post-discharge by a healthcare provider is always appreciated, as it makes them feel cared for and safer with medication use. Mostly, patients mentioned that a home visit post-discharge, which they received during their participation in the pharmacy-led transitional care program, [26] is a facilitator, especially when changes are conducted in the pharmacotherapy. They felt that this visit clarified medication use and management, and led to practical advice adjusted to their own needs:

*“Just to monitor the home situation, see how all the various medication is used and taken, how they are dealt with and how they are stored. I think that is also, it may be very helpful if that is thoroughly examined.” (Male, 73 years old, cardiology)*

Moreover, patients perceived a home visit as additional support for their therapy and found this additional attention pleasant:

*“It is quite a reassurance that this is done; it means the continuation of hospital care at home is well-organized … it is given attention, which is really great.” (Male, 79 years old, pulmonology)*

Patients emphasized that this could also lower the threshold for patients to ask questions regarding medication. Some patients, however, felt that they were being monitored during the home visit in the way that they were using or managing their medication: *“The idea that the pharmacist really came to check if you were not messing up the medication, and if I could figure it all out. He was not only there as, say, a support, he was really looking whether I took my medication each time.”(Male, 75 years old, cardiology).*

## Discussion

This qualitative study provides insight into perspectives of patients on barriers and facilitators of medication use, during the transition from hospital to home. Barriers included a lack of personalized care, insufficient information transfer between healthcare providers, and problems in the organization of healthcare. Many practical solutions were suggested by patients to overcome the identified (sub) themes of barriers, of which 2 main themes of facilitators were identified: a personal medication-counsellor in the care continuum and post-discharge follow-up care by qualified healthcare providers.

Overall, the findings of the study indicate that patients do not feel that care is personalized to their preferences, needs or functional status. As the study results show, many patients have difficulty using their medication adequately due to cognitive impairment, or because they encounter difficulties with medication adherence as they receive insufficient medication management tools or (social) support. This can ultimately result in MRPs post-discharge, as shown by previous studies [5, 6, 32]. The introduction of personalized care could be promising to facilitate safe medication use in the care continuum, as it has already shown improvements in people’s physical and psychological health status, and patients’ capability to self-manage their condition when compared to usual care [33–35]. However, according to Hesselink et al. [18]*,* providing personalized care during hospitalization is often difficult for healthcare providers because of the hurried discharge processes, which are often not well prepared, and incorrect estimations of patients’ capabilities and informational needs. To that end, patients in this study suggested that an assessment should be made by healthcare providers during hospitalization, to evaluate which patients are in need of extra care. Those patients should be appointed a personal medication-counsellor, who provides care tailored to their needs and helps to overcome barriers during transitions in care. It does not matter to patients which professional fulfils this role, as long as this person is closely related to the patient and could act as a point of contact between his or her involved healthcare providers in the care continuum. Introducing a qualified nurse as a ‘transition coach’ during hospital discharge has been found to reduce hospital readmission rates in chronically ill patients [36]. Although promising, this study did not investigate the effect on the occurrence of post-discharge MRPs, such as adverse drug events, which are known to affect patients’ health and quality of life [3, 5, 13, 37]. Another effective intervention is the provision of post-discharge follow-up care, such as home visits [38–40]. Nearly all patients in this study widely appreciated the home visits, as they received support on medication use and practical advice adjusted to their own needs (e.g. dose-dispensing systems for patients who regularly forget to take their medication). On the other hand, patients acknowledged that this service would most likely be expensive and moreover, not necessarily needed for everyone or after every hospitalization. Thus, finding the right balance between the provision of care by healthcare providers and inquiring patients’ needs in care transitions is essential.

In addition to a lack of personalized care, patients also experienced inadequate transfer of changes in pharmacotherapy, which is a known problem and has been linked to increased MRPs and unplanned readmissions several times [5, 6, 9, 15, 16]. These communication breakdowns had an impact on patients’ trust in their healthcare provider as they feared errors in their medication overviews due to incorrect handovers. Proposed facilitators to address this barrier included enhancing relationships between primary and secondary healthcare providers, by organizing frequent gatherings to discuss patients’ potential MRPs. Also, some patients mentioned the engagement of patients as active participants, which results in safer handover communication and lower re-hospitalization rates [36, 39].

Finally, the study findings elucidate obstacles patients perceive with the organization of processes in the hospital, specifically, the substitution of branded and generic medication. In the Netherlands, formulary agreements, specifying the type and brand of medication approved for prescribing, often differs between primary and secondary healthcare settings. Hospital (outpatient) pharmacies often supply the branded version (due to discounts for branded medication), whereas community pharmacies generally dispense generic medications. The brand and generic drug, however, may appear completely different to the patient due to different packages and pill appearance, which can result in decreased adherence to and persistence of medication use [40]. To address this barrier and avoid confusion with medication use, patients find it important to be informed about their changed medication, both verbally and by written communication [9].

Altogether, perceived barriers by patients could be unknown or invisible to healthcare providers as patients’ assessment of quality in care is not always aligned with that of healthcare providers [41]. Therefore, to reduce post-discharge MRPs, future research into perspectives of healthcare providers on barriers and facilitators during the transition from hospital to home is needed as well.

### Strengths and limitations

The primary strength of this study is the identification of barriers and facilitators specifically for medication use from a patient perspective, which could help with the development of effective interventions to reduce MRPs. Furthermore, identified barriers were schematically visualized using a fishbone diagram. This analytical tool has already been used in several studies to generate solutions to or identify causes of a quality of care problem [42, 43].

However, some limitations should be acknowledged. First, patients were selected from the intervention group of a larger study which can influence the study results, as patients already knew some facilitators throughout the received interventions. This is especially notable for patients perceiving a home visit as a facilitator. It is possible that other (sub) themes would have been identified if focus groups had been performed with patients who did not receive any intervention. However, patients have difficulties to reflect on their needs if they are not queued about the possibilities [10] and therefore, we believe that the selection of the intervention group provided a better insight into barriers and facilitators, as patients reflected on previous experiences with transitions in care. Second, because a relatively small group of patients from the intervention group participated, it might be possible that selection bias occurred. Nonetheless, the demographic characteristics were essentially similar in both groups, indicating qualitative clarity. Furthermore, no new themes emerged in the third focus group and therefore, data can be considered to be comprehensive. Also, according to literature, a sample size of 2 to 3 focus groups will likely capture at least 80% of themes on a topic—including those most broadly shared—in a study with a relatively homogeneous population using a semistructured guide [44] which was the case for our focus groups. In addition, two comparable qualitative research studies on this topic, interviewed 13–19 patients and also reported considerable agreement between participants [24, 45].

Finally, some barriers are related to the local Dutch organization of care and could therefore differ between settings and countries. However, the identified themes, especially the barriers, are similar to what other studies have found [46] e.g. communication barriers, medication dispensing problems, which in our opinion indicates that these study results are not only relevant to locally situated patients or healthcare providers. Still, future research should be conducted to replicate our findings.

## Conclusions

During transitions from hospital to home patients experience individual-, healthcare provider- and organization level barriers that can lead to MRPs post-discharge. To reduce these problems, the care provided should be more personalized, taking patient’s values, preferences and needs into account. Future research should focus on personal-medication counselors in the care continuum and post-discharge follow-up care as it may overcome communication, emotional, information and organization barriers with medication use.

## Additional files

Additional file 1: Example list of barriers and facilitators with medication use during the transition from hospital to home. (DOCX 21 kb)

Additional file 2: **Table S3**: Demographic characteristics of each focus group participant. (DOCX 21 kb)
