# Supplementary material for: Barriers and facilitators with medication use during the transition from hospital to home: a qualitative study among patients
Source: BMC Health Serv Res. 2019 Mar 29;19:204. doi: 10.1186/s12913-019-4028-y (PMC6441233; doi:10.1186/s12913-019-4028-y)
Supplement: Supplementary file 1 — Example list of barriers and facilitators with medication use during the transition from hospital to home. (DOCX 21 kb) [file 12913_2019_4028_MOESM1_ESM.docx]

**Example list of barriers**

1. Symptoms/side effects
   - Experiencing side effects due to changes in medication implemented during hospitalization
2. Practical problems
   - Problems with adjusting the medication regimen to the daily schedule
     1. Trouble with telling medication apart (when using multiple medication)
   - Logistic problems
     1. Regularly running out of medication
     2. Often expired medication
     3. Storage of medication
     4. Doubling-up of medication (generic and brand-name)
   - Difficulty with handling the immediate packaging and pressing the medication out
   - Problems with reading and/or understanding the instructions for use
   - Difficulty with completing preparation of medication before use and administration:
     1. Inhaler technique
     2. Instilling eye or ear drops
     3. Injections
     4. Applying medication patches or ointment
3. Information/knowledge
   - Poor knowledge regarding medication indication or implemented changes
   - Lack or incorrect information transfer regarding:
     1. Indication
     2. Effect
     3. Side effect
     4. Use
     5. Interactions
     6. Contraindications
4. Supervision/support
   - Insufficient or lack of support with medication use (e.g. administration or preparation of medication)
   - Doubts about medication (effect, fear of side effects)
   - Limited health literacy
5. Communication
   - Communication with the patient
     1. Who patients should contact when experiencing medication-related problems.
   - Communication among healthcare providers
     1. Who is responsible for taking care of the patient (e.g. adequate and complete information transfer)

**Example list of facilitators**

1. Symptoms/side effects
   - Information on most common side effects of changed medication (implemented during hospitalization):
     1. Which side effects can patients expect
     2. Are the side effects temporary
     3. How long do the side effects last for
     4. In which case should a healthcare professional be contacted
     5. Which healthcare professional should be contacted
2. Practical problems
   - Daily medication schedule
   - Electronic (repeat) prescription service
   - Dosing aids for administration problems
   - Dose-dispensing systems
3. Information/knowledge
   - Verbal and written communication about medication:
     1. effect
     2. side effects
     3. interactions
     4. contraindications
   - Instructions on how to use medication:
     1. Inhaler instructions
     2. Self-administration of insulin
4. Supervision/support
   - Home healthcare service
   - Informal caregiver/family
   - Post-discharge home visit or telephone call

5. Communication

- Guidelines about who patients or healthcare professionals should contact in case of medication-related problems or questions about medication:
  - For questions about hospital medication the physician or clinical pharmacy should be contacted
  - For other medication related questions the general practitioner or community pharmacist should be contacted
