# Supplementary material for: Barriers and facilitators with medication use during the transition from hospital to home: a qualitative study among patients
Source: BMC Health Serv Res. 2019 Mar 29;19:204. doi: 10.1186/s12913-019-4028-y (PMC6441233; doi:10.1186/s12913-019-4028-y)
Supplement: Supplementary file 2 — Table S3: Demographic characteristics of each focus group participant. (DOCX 21 kb) [file 12913_2019_4028_MOESM2_ESM.docx]

| **Gender** | **Number of medicationat discharge** | **Number of in-hospital medication changes** | **Help with medication use at home** | **Living situation** | **Hospital** | **Type of admission** | **Ward** |
| --- | --- | --- | --- | --- | --- | --- | --- |
| Male  **Focus group 1** | 15 | 3 | No | Living together | OLVG | Planned | Cardiology |
| Male | 9 | 1 | No | Living together | BovenIJ | Planned | Cardiology |
| Female | 6 | 3 | No | Living together | BovenIJ | Unplanned | Internal medicine |
| Male | 7 | 5 | Yes | Living together | BovenIJ | Unplanned | Neurology |
| Female | 10 | 2 | No | Alone | BovenIJ | Unplanned | Cardiology |
| Female | 10 | 4 | No | Living together | BovenIJ | Unplanned | Internal medicine |
| Male | 12 | 2 | No | Alone | BovenIJ | Unplanned | Cardiology |

| Male  **Focus group 2** | 7 | 1 | No | Living together | OLVG | Unplanned | Cardiology |
| --- | --- | --- | --- | --- | --- | --- | --- |
| Male | 6 | 4 | Yes | Living together | OLVG | Unplanned | Cardiology |
| Male | 16 | 6 | Yes | Alone | OLVG | Unplanned | Pulmonology |
| Female | 5 | 2 | No | Living together | OLVG | Unplanned | Cardiology |
| Male | 7 | 6 | Yes | Living together | OLVG | Unplanned | Cardiology |
| Female | 8 | 2 | No | Alone | OLVG | Unplanned | Internal medicine |
| Female | 10 | 4 | Yes | Alone | OLVG | Unplanned | Internal medicine |

**Focus group 3**

| Male | 11 | 2 | No | Alone | OLVG | Unplanned | Cardiology |
| --- | --- | --- | --- | --- | --- | --- | --- |
| Male | 13 | 7 | No | Living together | OLVG | Unplanned | Pulmonology |
| Female | 9 | 7 | No | Living together | OLVG | Unplanned | Cardiology |
| Male | 13 | 2 | No | Living together | OLVG | Unplanned | Internal medicine |
| Female | 8 | 3 | No | Living together | BovenIJ | Unplanned | Internal medicine |
